# Exploring microRNA targeting as a promising approach for solid tumor treatment

**DOI:** 10.3389/fonc.2025.1570093

**Published:** 2025-07-21

**Authors:** Behrouz Shademan, Vahidreza Karamad, Alireza Nourazarian, Cigir Biray Avci

**Affiliations:** ^1^ Medical Journalism, School of Paramedical Sciences, Shiraz University of Medical Sciences, Shiraz, Iran; ^2^ Department of Medical Biology, Ege University Medical School, Izmir, Türkiye; ^3^ Department of Basic Medical Sciences, Khoy University of Medical Sciences, Khoy, Iran

**Keywords:** microRNA, solid tumors, cancer therapy, miRNA biology, tumor microenvironment

## Abstract

The discovery of microRNAs (miRNAs) and their pivotal role in gene regulation has opened up new avenues for innovative cancer treatments. Recent years have witnessed extensive research into the intricate mechanisms of miRNAs and their impact on solid tumors. These small non-coding RNA molecules are central to gene regulation and are frequently dysregulated in various cancers, particularly solid tumors. Dysregulation of specific miRNAs can initiate, progress, and metastasize tumors, making them appealing targets in cancer therapy. This article explores recent studies on identifying specific miRNAs associated with solid tumors and their influence on crucial signaling pathways. These findings enable precise targeting of cancer cells, reducing damage to healthy tissues and minimizing side effects commonly associated with conventional cancer treatments. Understanding the complex regulatory networks governed by miRNAs allows researchers and clinicians to develop highly effective, personalized treatment strategies, heralding a new era of tailored cancer medicine. Ongoing research in this field holds immense promise for pioneering targeted therapies that can significantly improve outcomes and the quality of life for individuals battling solid tumors.

## 1 Introduction

The initiation of tumorigenic processes is pivotal in converting normal cells, potentially fostering malignancy. Comprehending the regulatory mechanisms linked to the onset and progression of different cancers carries significant clinical implications, including early prevention, precise screening, and personalized treatment strategies. MicroRNAs (miRNAs) have been a focus of extensive research for decades among the factors influencing altered gene expression in carcinogenesis (1). MiRNAs, typically around 22 nucleotides in size, are small noncoding RNAs that serve as post-transcriptional regulators, finely adjusting the coding efficiency of messenger RNA (mRNA) (2). These miRNAs interact with the RNA-induced silencing complex (RISC), composed of single-stranded miRNA, Argonaute, and GW182 proteins, leading to either the inhibition of translation or the breakdown of the specific regulatory target (3). The regulation carried out by miRNAs contributes to cellular responses under various stress conditions, including nutrient deprivation, oxidative damage, low oxygen (hypoxia), and DNA injuries, and is thus associated with the progression of malignant disorders (4). As a result, dysregulation of miRNA expression has been demonstrated to exert a bidirectional effect on oncogenesis or tumor suppression (5).

Recent studies indicate a notable connection between miRNA expression and epigenetic regulation, particularly regarding the methylation of CpG islands within promoter regions associated with cancer (6). The inactivation of miR-127, miR-124-1, or miR-129-2 is closely associated with hypermethylation of CpG island-containing promoters in several solid cancer types (7–9). Furthermore, anomalies in miRNA processing components, such as Drosha or the DGCR8 protein, are frequently detected in various malignancies (10). While the exact role of Drosha or DGCR8 in carcinogenesis is still debated, disruptions in miRNA processing machinery are strongly correlated with a comprehensive change in the miRNA expression profile (11). Mutations in the Dicer gene are associated with DICER1 syndrome, a condition that increases the susceptibility of affected individuals to various cancers (12). The presence of mutant Dicer protein interferes with the biogenesis of miRNAs and modifies gene expression patterns (13). Recent research has revealed that miRNA genes are often found in chromosomal areas susceptible to copy number variations associated with cancer (14, 15). Genomic instability caused by cancer may result in the amplification or loss of miRNA gene regions, causing changes in the number of miRNA copies (16). Based on existing evidence, we have compiled a summary of the current knowledge regarding the role of miRNAs in the development of common solid tumors such as colorectal, lung, breast, and liver cancers. Additionally, this review discusses the benefits and challenges of using miRNAs as therapeutic agents in cancer treatment.

## 2 Barriers to current cancer treatments

**Figure 1:**
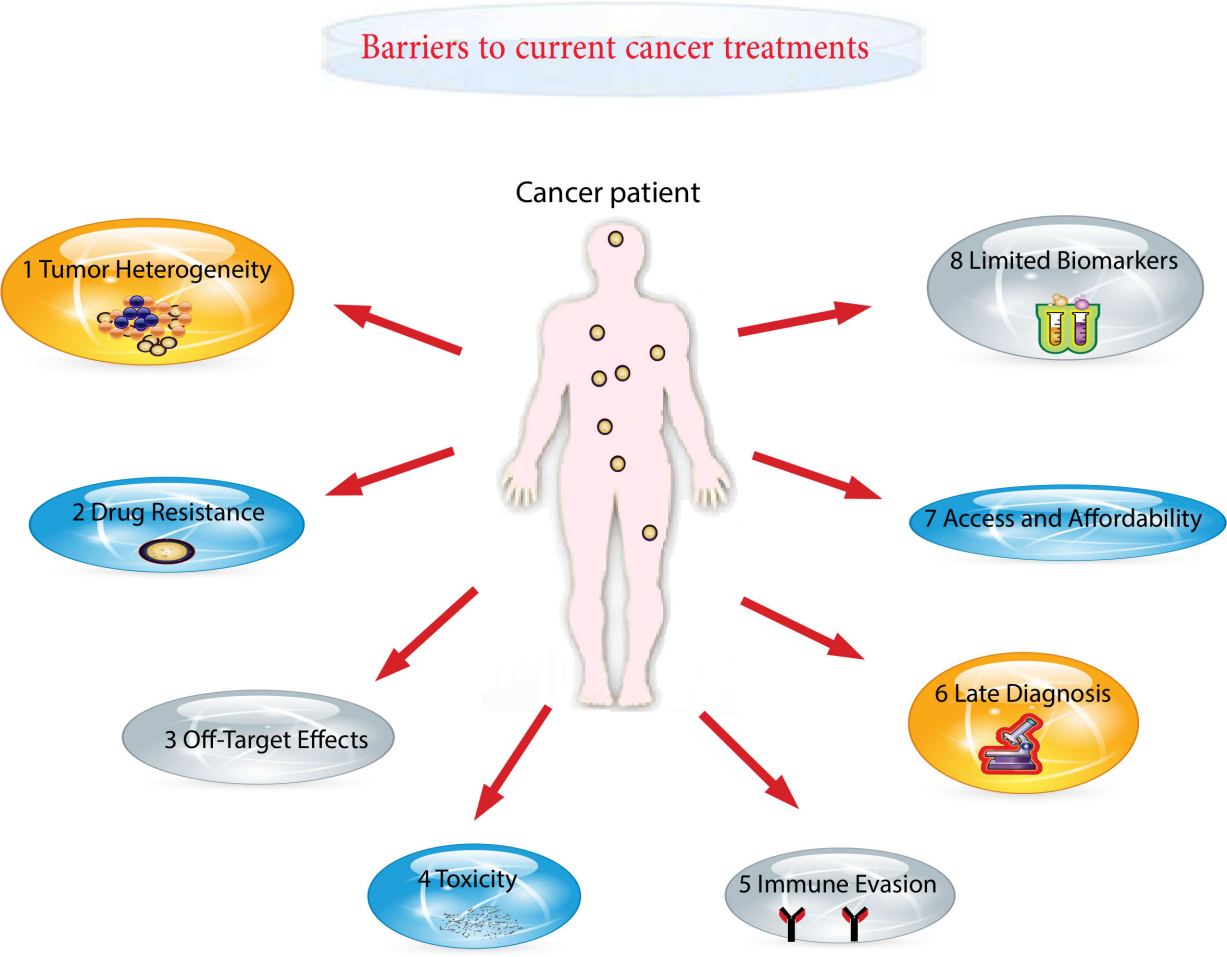
Some of the major barriers to current cancer treatments in healthcare delivery.

Cancer remains the foremost cause of death globally, responsible for close to 10 million fatalities in 2020 (17). Alarmingly, around 70% of cancer-related deaths take place in low- and middle-income countries, which receive less than 5% of the global resources allocated for cancer control (18). This disparity is further exacerbated by the lack of reliable cancer registries covering around 85% of the global population, leading to an underestimation of the actual burden (19). In many resource-limited settings, fragile healthcare systems and high out-of-pocket costs pose significant barriers to accessing timely and effective cancer care (20). To address these challenges, a patient-centered approach has gained attention, emphasizing individualized care plans based on patient’s values, preferences, and overall life context (21). However, successful implementation requires patients to have sufficient psychosocial and financial resources—an expectation not met in many underserved populations (22, 23). Consequently, many individuals are dying from cancers that are otherwise preventable or treatable, resulting in a significant decline in quality of life. Evidence indicates that those from socioeconomically disadvantaged backgrounds are disproportionately affected and tend to: firstly, experience delays in initiating treatments such as surgery, systemic therapy, or radiotherapy; secondly, have lower likelihoods of receiving any form of cancer treatment; thirdly, demonstrate reduced adherence to treatment protocols including chemotherapy, hormone therapy, biologic therapy, or immunotherapy; fourthly, receive care in non-specialized settings; and fifthly, undergo treatment that diverges from established clinical guidelines, in contrast to their more privileged counterparts (24–26) (**Figure 1**). These structural and systemic barriers highlight the urgent need for innovative, accessible, and targeted treatment strategies, such as miRNA-based therapeutics, which may offer scalable and less resource-intensive alternatives for improving global cancer care outcomes.

## 3 MicroRNAs

miRNAs are short RNA molecules that do not code for proteins but play a crucial role in regulating gene expression. Their biogenesis involves multiple complex steps. First, miRNA genes are transcribed by RNA polymerase II or III, producing a long primary transcript called pri-miRNA (27). This pri-miRNA features a 5’ cap and a poly-A tail, forming a structured molecule. The enzyme Drosha, aided by DGCR8, processes the pri-miRNA by cleaving it into a shorter precursor called pre-miRNA. This pre-miRNA has a characteristic hairpin shape and is about 60 to 100 nucleotides long (28, 29). Pre-miRNAs are shuttled from the nucleus to the cytoplasm via the coordinated action of Ran GTP and exportin-5. Once in the cytoplasm, Ran GTP is hydrolyzed to Ran GDP, which triggers the release of pre-miRNA from exportin-5 (30).

**Figure 2:**
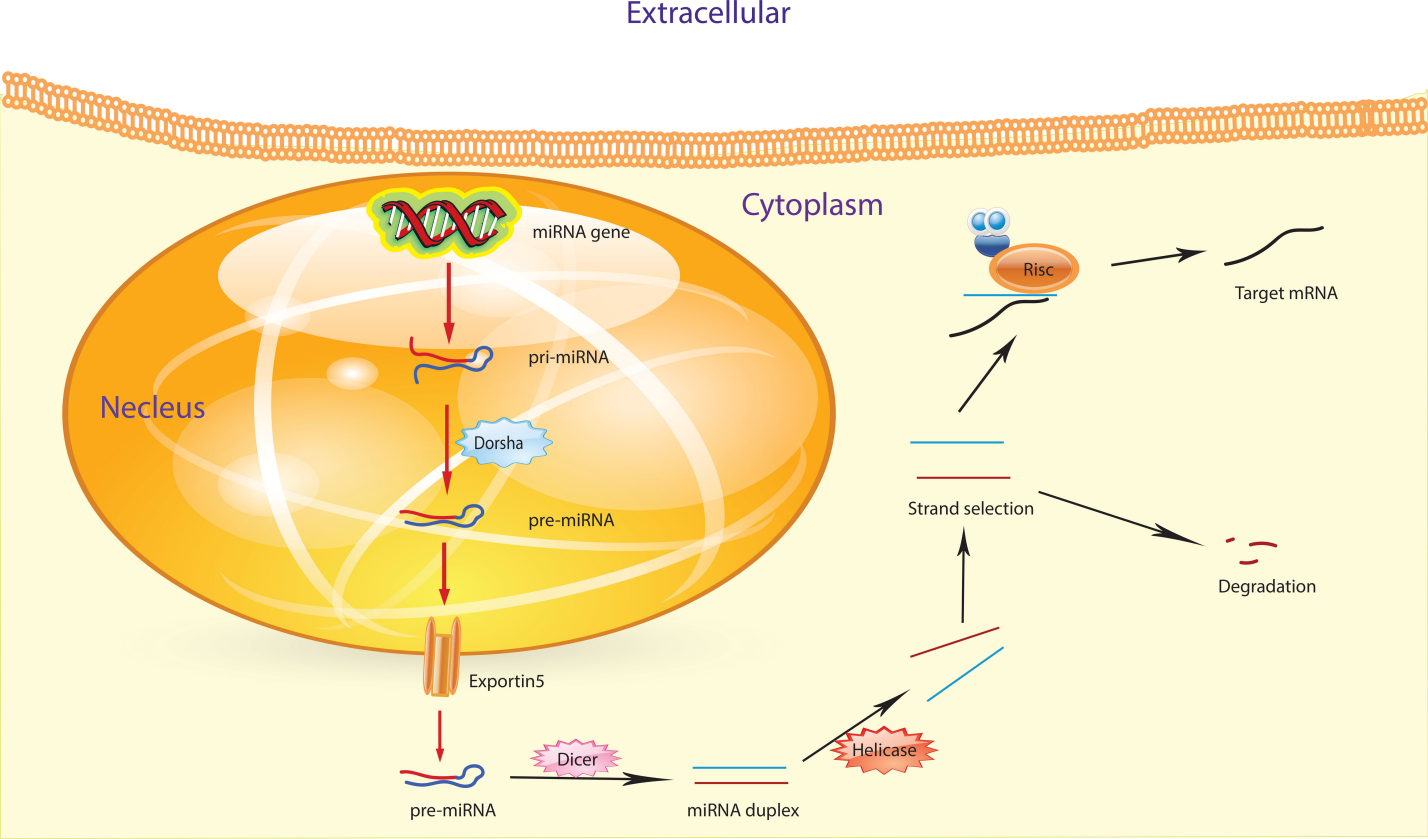
The figure illustrates the process of miRNA biogenesis. Inside the nucleus, pri-miRNA undergoes a transformation initiated by the RNase III endonuclease Drosha, aided by its cofactor Dgcr8, resulting in the formation of smaller stem-looped structures known as precursor miRNA (pre-miRNA). These pre-miRNAs are then transported from the nucleus to the cytosol by exportin 5. Further processing takes place in the cytosol, where a second RNase III enzyme, Dicer, collaborates to produce mature miRNA.

In the cytoplasm of the cell, Dicer, an enzyme classified as a ribonuclease III, collaborates with a protein to cleave pre-miRNA, resulting in the production of mature miRNA. The resultant double-stranded mature miRNA (miRNA/miRNA*) typically spans approximately 22 nucleotides and lacks a circular structure (30, 31). Within this miRNA/miRNA* duplex, there may be unpaired bases and incomplete bonding between the two strands. Subsequently, one of the duplex miRNA strands, either miRNA or miRNA*, integrates into the RISC complex, while the other strand undergoes cleavage or degradation. The RISC complex assumes a critical role in guiding fully formed miRNA to its specific target mRNA, thereby inhibiting the translation process and regulating protein synthesis (**Figure 2**) (32, 33). The discovery of miRNAs has unveiled that approximately 1% of the human genome participates in miRNA coding. Additionally, each miRNA can regulate up to 200 mRNAs (34). Understanding the functions and origins of miRNAs is indispensable for comprehending their involvement in various biological processes such as development, cell differentiation, and diseases. To probe into the functions of miRNAs, it is essential to identify their target genes and grasp the biological pathways in which they are involved. Various methods, including microarray analysis and RNA sequencing, can be employed for this purpose (35).

Considering the significant impact miRNAs have on gene expression and their participation in various cellular processes, it’s understandable that irregularities in miRNAs are linked to diverse pathological conditions. Abnormal miRNA expression patterns have been observed in a wide array of human cancers (36, 37). These deviations stem from different genomic abnormalities, including aberrant miRNA biogenesis and altered epigenetic regulation.

## 4 miRNAs in solid tumors

The onset of tumorigenic processes triggers the conversion of normal cells, potentially leading to malignancy. Understanding the regulatory mechanisms linked to various cancers is crucial for clinical applications, including early prevention, precise screening, and personalized treatments. MicroRNAs (miRNAs) emerge as significant regulatory factors influencing altered gene expression in carcinogenesis, extensively studied for decades (1). Disruptions in miRNA expression can arise through various pathways, encompassing transcriptional regulation, epigenetic methylation of miRNA-containing sites, the miRNA processing pathway, and interaction with long non-coding RNAs serving as miRNA sponges (38, 39). MiRNAs with negative modulation facilitate the generation of numerous mRNA transcripts while meticulously regulating protein production (40). Alterations in the miRNA expression profile are noticeable in human cancers, where certain miRNAs are markedly overexpressed or lost in tumors compared to normal tissues. Oncogenic miRNAs, termed oncomiRs, bolster tumor growth by suppressing tumor-suppressing genes, whereas tumor-suppressing miRNAs, referred to as anti-oncomiRs, target oncogenes to impede tumor progression (41). The diminished activity of miR-127, miR-124-1, or miR-129-2 strongly correlates with excessive methylation of CpG island-containing promoters in various solid cancers (9, 42). Furthermore, proper functioning and expression of miRNA processing components like Drosha or the DGCR8 protein are commonly disrupted in various malignancies (43). While the impact of Drosha or DGCR8 on cancer development remains contentious, the disruption of miRNA processing machinery is closely associated with widespread alterations in miRNA expression patterns (11). Recent research indicates that miRNA loci often reside within chromosomal regions prone to cancer-related copy number variations (CNV) (44). Genomic instability induced by cancer results in the amplification or deletion of miRNA loci, leading to changes in miRNA copy numbers (16). Drawing from the accumulated evidence, we have compiled an overview of the existing knowledge regarding the influence of miRNA on the development of common solid cancers, including pancreatic cancer, colorectal cancer, breast cancer, lung cancer, hepatocellular carcinoma, ovarian cancer, and glioma. Furthermore, this review assesses the benefits and challenges associated with the utilization of miRNA in cancer treatment strategies.

### 4.1 miRNAs targeting metastatic genes

The exploration of gene signatures and biomarkers, including miRNAs, for predicting metastasis outcomes in patients, is still in its nascent stages. The first major discovery was made by detecting abnormal miRNA expression in human B cell chronic lymphocytic leukemia (CLL) through a microarray equipped with a wide range of precursor and mature miRNA probes (45). Subsequently, miRNAs detected in the bloodstream have been investigated as potential biomarkers for diagnosing and prognosing various diseases, including cancers. Notably, the miRNAs found to be deregulated in cancer have been revealed to function as oncogenes or tumor suppressors, acting by inhibiting specific target genes (46). miR-21, the pioneering “oncomiR” discovered, was found to play a crucial role in promoting Epithelial-Mesenchymal Transition (EMT). In invasive breast cancer cells (MDA-MB-231), inhibiting miR-21 with antagomir successfully reversed both EMT and the cancer stem cell (CSC) characteristics. This reversal occurred due to increased expression of PTEN, leading to the deactivation of AKT/ERK pathways (47). Another significant miRNA, miR-9, induced by MYC/MYCN, was proven to directly target E-cadherin, thereby promoting metastasis in breast cancer (48). Clinical observations revealed a significant rise in miR-9 levels in primary breast tumors of patients who later developed metastasis, compared to those who remained metastasis-free (48). Additionally, miR-29a was demonstrated to trigger EMT in Ras-transformed mouse mammary epithelial cells by regulating the expression of TTP (tristetraprolin) (49). In a study focused on colon cancer, researchers found that PROX1 (Prospero homeobox 1) activated the expression of miR-9, subsequently leading to the downregulation of E-cadherin (50). Apart from the process of EMT (Epithelial-Mesenchymal Transition), there exists a reverse phenomenon known as MET (Mesenchymal-Epithelial Transition), crucial for metastatic cells to colonize distant organs. Recent studies have highlighted the significance of miRNA regulation in MET transition. For instance, Chen and colleagues demonstrated that miR-103/107 directly targets the MET inducers KLF4 and DAPK, thereby promoting metastasis (51). These results highlight the crucial involvement of miRNAs in the MET of cancer cells.

### 4.2 miRNAs targeting apoptotic genes

Cathepsins are a family of lysosomal proteolytic enzymes that, when released into the cytoplasm, trigger apoptosis (52). They are essential in both the intrinsic apoptosis pathway—by controlling the release of pro-apoptotic factors from mitochondria—and the extrinsic pathway—by suppressing apoptosis inhibitors (IAPs). Among these enzymes, cathepsin S (CTSS), a cysteine protease, has been linked to tumor development and chemotherapy outcomes in colorectal cancer (CRC) patients (53). Studies suggest that cathepsin S promotes tumor invasion through degradation of the extracellular matrix and the release of matrix-derived growth factors, which in turn enhance angiogenesis (54).

MiRNAs play a key role in numerous cellular functions, including the regulation of apoptosis. Specific miRNAs—such as miR-124, miR-195, miR-148a, miR-365, miR-125b, miR-129, miR-143, and miR-203—have been shown to influence apoptosis by targeting genes like BCL2 and PUMA, which is a pro-apoptotic protein within the Bcl-2 family (55). Furthermore, CTSS, in addition to blocking Bcl-2 family members, inhibits IAPs. Specifically, miR-203a directly targets BCL2, resulting in reduced BCL2 expression and activation of the intrinsic apoptosis pathway (55). BCL2 has also been studied in conjunction with various other miRNAs, including miR-491, miR-143, miR-148a, miR-365, miR-1915, miR-204, and miR-125b (55). These miRNAs could potentially serve as crucial targets for therapeutic interventions due to their evident role in regulating apoptosis.

### 4.3 miRNAs in tumor epigenetics

The epigenetic system consists of intricate gene regulatory mechanisms that operate at the chromatin level without modifying the DNA sequence itself. These regulatory processes include DNA methylation, histone post-translational modifications, incorporation of histone variants, changes in nucleosome spacing and density, three-dimensional chromatin structure, and the influence of non-coding RNAs (56). The dynamic and intricate regulation of epigenetic mechanisms is crucial in carcinogenesis, tumor progression, and therapy resistance. Small noncoding RNAs, including microRNAs (miRNAs) and long noncoding RNAs (lncRNAs), modulate the activity of key enzymes such as DNA methyltransferases (DNMTs), which suppress gene expression by adding methyl groups to DNA, and histone deacetylases (HDACs), which facilitate chromatin condensation and transcriptional repression by removing acetyl groups from histones (57, 58). MicroRNAs modulate gene expression patterns by regulating the activity of epigenetic enzymes such as DNA methyltransferases and histone deacetylases, leading to the silencing or activation of tumor suppressor genes, thereby influencing cancer development and progression (59, 60).

MiRNAs influence histone-modifying enzymes by directly binding to the mRNAs of these targets, thereby modulating their expression and affecting epigenetic regulation. Moreover, several miRNAs are known to directly interact with proteins responsible for histone modifications and broader epigenetic regulation. For instance, miR-29a targets the MYC/HDAC3/EZH2 axis in lymphoma, while miR-200a regulates HDAC4 and SP1 in hepatocellular carcinoma. Similarly, miR-224 influences the expression of HDAC1, HDAC3, and EP300 in the same cancer type. Additional examples include miR-212, which modulates EZH2, G9a, and HDACs in lung cancer; miR-126, targeting HDAC2 in prostate cancer; and miR-34a, which regulates SIRT1 in breast cancer. MiR-34b affects HDAC1, HDAC2, and HDAC4 in prostate cancer, while miR-127, miR-411, miR-431, and miR-432 are implicated in HDAC regulation in osteosarcoma. Other miRNAs include miR-9-5p (HDACs in gastric cancer), miR-101 (EZH2 in glioblastoma), miR-22 (TIP60 and HDAC4 in breast cancer), and miR-125 (HDAC4 and HDAC5 in breast cancer). Additionally, miR-142 targets ASH1L/KMT2H in leukemia and thyroid cancer, miR-675 regulates SUV39H2/KMT1B in liver cancer, miR-122 influences SUV39H1/KMT1A in hepatocellular carcinoma, and miR-101 modulates KMT6/EZH2 in non-small cell lung cancer (NSCLC), prostate, and renal cancers. Lastly, miR-195 targets PRMT4/CARM1 in colorectal cancer, and miR-155 regulates JMJD1A in nasopharyngeal carcinoma (61). Studies have shown that miR-449a suppresses HDAC-1 expression, indicating that this microRNA may regulate prostate cancer cell growth and survival through this mechanism (62). In the context of cancer, many miRNAs are subject to regulation through histone methylation, creating intricate feedback loops between miRNA activity and epigenetic methylation mechanisms. Increased expression of specific miRNAs—such as miR-101, miR-125a-5p, miR-122, miR-675, miR-212, miR-22-3p, miR-142, and miR-181a—has been shown to influence the activity of histone methyltransferases. These interactions can significantly alter chromatin structure and subsequently affect the transcriptional landscape of cancer cells (61). Recognizing specific miRNAs that engage with histone-modifying enzymes opens new avenues for the development of more precise and targeted cancer treatment strategies (63, 64). For instance, miR-29b, a tumor suppressor, targets HDAC4 and forms a regulatory feedback loop; silencing HDAC4 reduces multiple myeloma cell survival and promotes apoptosis and autophagy (65). In nasopharyngeal cancer, downregulation of miR-129 contributes to resistance against SAHA, while restoring miR-129 expression overcomes this resistance. The lncRNA NEAT1 regulates miR-129 and the miR-129/Bcl-2 axis, further influencing SAHA tolerance (66).

miRNA genes are often epigenetically regulated through DNA methylation and histone modifications, affecting their expression in diseases like cancer. Nearly half are located near CpG islands, and some of them are susceptible to methylation-induced silencing in various tumors (67). Additionally, methylation also plays a role in controlling the expression of genes involved in miRNA biogenesis (68). In 2007, the earliest reports highlighted the association between DNA methylation and the control of let-7 miRNA family expression. Lu et al. (69) reported that decreased expression of the tumor-suppressive let-7a-3 correlated with increased methylation, which influenced insulin-like growth factor-II expression and affected ovarian cancer patient survival. Likewise, epigenetic silencing of miR-125a due to hypermethylation has been observed in multiple myeloma, colorectal cancer, and gastric cancer (59). Notably, the histone methyltransferase SUV39H1 was identified as a target of miR-125a in GC, and epigenetically silenced miR-125a-5p was shown to self-reactivate by targeting this methyltransferase (70). Moreover, diminished expression of miR-98 in glioma tissues and cell lines has been associated with elevated levels of DNA methylation, which in turn is linked to greater tumor aggressiveness, increased invasive capacity, and reduced patient survival rates (71). Hypermethylation of MiR-15a/b and miR-16 clusters has been linked to the progression of myelodysplastic syndrome into acute myeloid leukemia and is associated with poor prognosis (72). The miR-497~195 cluster also shows promoter methylation in breast cancer, where forced expression of these miRNAs reduces cell proliferation and invasion by targeting genes such as RAF-1, CCND1, and mucin-1 (59). Similar inhibitory effects of miR-497 were reported in gastric cancer cells (73). Significant methylation-driven downregulation of miR-195 has been reported in pancreatic cancer, where treatment with 5-Aza-CdR restored its levels and suppressed proliferation, migration, invasion, and epithelial-mesenchymal transition of PC cells (72). MiR-424 functions similarly as a tumor suppressor, showing an inverse correlation between its expression and promoter DNA methylation in glioblastoma, cervical cancer, endometrial endometrioid adenocarcinoma, and ovarian cancer (59).

Overall, miRNAs act as epigenetic modulators that can reverse the silencing of key regulatory genes, thus contributing to tumor suppression or, in some contexts, tumor promotion. Understanding these interactions provides new insights into cancer biology and highlights potential epigenetic-based therapeutic targets in oncology.

### 4.4 The interplay between miRNAs and tumor microenvironment

Immune cells present in the tumor microenvironment (TME) have been found not only to lack effective anti-tumor activity but also to potentially promote tumor development. MiRNAs have emerged as crucial molecular players facilitating communication between tumor cells and immune cells within the TME. The primary immune cell types involved include macrophages, myeloid-derived suppressor cells, dendritic cells, and natural killer cells (74). Recent studies have highlighted the pivotal role of miRNAs in regulating the function of tumor-associated macrophages (TAMs) and cancer progression (74). miR-100c is highly expressed in tumor-associated macrophages (TAMs) and supports the maintenance of their phenotype by regulating the mTOR signaling pathway. Blocking this miRNA in breast cancer models notably decreased the metastatic and invasive abilities of tumor cells, highlighting its involvement in cancer progression (75). miR-375, a context-dependent miRNA, is downregulated in hepatocellular carcinoma and gastric cancer but upregulated in breast cancer. It can be taken up by macrophages via the CD36 receptor on their surface, and by targeting TNS3 and PXN, it alters macrophage infiltration and migration (76). Macrophage polarization plays a crucial role in tumor development. While M1 macrophages are associated with pro-inflammatory and anti-tumor activity, M2 macrophages exhibit anti-inflammatory and pro-tumor properties. Certain miRNAs, such as miR-145, can induce macrophage polarization toward the M2 phenotype in colorectal cancer, thereby promoting tumor progression (77). Certain miRNAs, including miR-1246, which are released by mutant p53 (mutp53) colon cancer cells, can reprogram macrophages, shifting them toward a phenotype that supports tumor growth (78). Conversely, miRNAs like miR-125b, miR-29, and miR-155 have been shown to shift macrophages from the M2 to the M1 phenotype, promoting anti-tumor activity (79–81). Specifically, in NSCLC models, macrophage-targeted delivery of miR-125b using HA-PEI nanoparticles significantly increased the proportion of M1 macrophages (82). In summary, miRNAs have multifaceted and intricate roles in influencing the traits, polarization, and activities of TAMs, serving as crucial regulators in both promoting and inhibiting tumor development.

Beyond affecting the phenotype and polarization of TAMs, miRNAs can indirectly influence tumor cell behavior by regulating the functions of these macrophages. TAMs have been shown to support tumor cell proliferation and invasion. For instance, TGF-β1 secreted by TAMs can increase VEGF expression, which in turn downregulates miR-34a, thereby enhancing the proliferation and invasiveness of colon cancer cells (83). Conversely, miR-98 has been found to inhibit TAM-mediated promotion of liver cancer cell metastasis and invasion by targeting and suppressing IL-10 expression (84). Tumor angiogenesis is essential for sustaining tumor growth, and TAMs significantly contribute to this process by producing angiogenic factors such as VEGF. Notably, under non-hypoxic conditions, elevated HIF-2α in TAMs induces pro-angiogenic genes like VEGFA and PDGFB. MiR-17 and miR-20a are involved in regulating this HIF-2α-driven angiogenesis in tumor cells (85). Collectively, these findings underscore the multifaceted roles of miRNAs in mediating TAM functions that shape tumor cell behaviors, including proliferation, invasion, angiogenesis, EMT, and stemness, further emphasizing their therapeutic potential.

### 4.5 MicroRNAs as regulators of immune checkpoint pathways

Immunotherapy, as one of the most advanced therapeutic approaches, has revolutionized the fight against cancer by boosting the body’s immune system. Unlike traditional therapies that directly target cancer cells, immunotherapy is designed to inhibit immune checkpoints, specifically by interfering with the interaction between programmed death protein 1 (PD-1) and its ligands such as PD-L1 (or PD-L2) (86). PD-1 is expressed on the surface of many immune cells, while PD-L1 is expressed by a variety of cell types, including cancer cells. The binding of PD-1 to PD-L1 inactivates T cells and attenuates the immune response. Increased PD-L1 expression is one of the known mechanisms for tumor immune evasion. Therefore, inhibition of the PD-1/PD-L1 pathway has been proposed as a promising approach with promising clinical applications in cancer therapy (87). miRNAs play a pivotal role in modulating immune checkpoint pathways, thereby influencing the tumor microenvironment and antitumor immunity (88). Tumor suppressor miRNAs play a crucial role in modulating the anticancer immune response by controlling immune checkpoints like PD-1, PD-L1, and CTLA-4. Some miRNAs help cancer cells evade immune detection by lowering their immunogenicity and weakening the immune response. Conversely, another set of miRNAs promotes the immune system’s ability to eliminate cancer cells. These immune-regulating miRNAs are referred to as im-miRNAs (89). While some miRNAs specifically target immune checkpoint proteins such as PD-1 or PD-L1, others can regulate both simultaneously.

miR-140 has been identified as a key regulator of PD-L1, with its expression significantly reduced in osteosarcoma. This microRNA also suppresses the mTOR signaling pathway, and its upregulation, when combined with mTOR inhibition, exerts a strong synergistic effect in suppressing tumor growth (90). Similarly, miR-15a and miR-15b function as tumor suppressors by binding to the 3′ untranslated region (3′-UTR) of PD-L1 mRNA, facilitating its degradation through the RISC. Laboratory studies have shown that both miRNAs enhance the cytotoxic activity of natural killer cells and CD8+ T cells against neuroblastoma cells. By targeting PD-L1 and reducing its expression, miR-15a and miR-15b trigger a strong antitumor immune response in neuroblastoma (91). Moreover, miR-43a functions as a suppressor of PD-L1 by lowering its mRNA expression, thereby exhibiting anti-tumor properties (92). In addition to decreasing PD-L1 levels, MRX34 has been shown to promote the infiltration of CD8+ T cells into tumors in cases of non-small-cell lung carcinoma (92). Further research indicates that combining MRX34 with radiotherapy can enhance the overall antitumor effectiveness (92).

In breast cancer tissues, increased levels of miR-21 have been associated with elevated PD-L1 expression. Studies in miR-21 knock-in mice revealed that treatment with radiotherapy or anti-PD-L1 antibodies enhanced apoptosis in both T cells and breast cancer cells. This was accompanied by decreases in CD3^+^CD8^+^ T cell populations, IFN-γ production, serum IL-2, tumor volume, and PD-L1 expression. Mechanistically, miR-21 upregulates PD-L1 in breast cancer cells by targeting PDCD4 through activation of the PI3K/Akt signaling pathway (93). Additionally, miR-101 and miR-222 within the tumor microenvironment influence the crosstalk between cancer-associated fibroblasts and tumor cells. The involvement of miRNAs in immune checkpoint regulation is further exemplified in non-small cell lung cancer, where miR-34 directly binds to the 3′-UTR of PD-L1 mRNA, resulting in its downregulation and highlighting the potential of miRNAs as biomarkers for immunotherapy response (94).

Additionally, miR-28 has been implicated in modulating T cell exhaustion by targeting multiple inhibitory immune checkpoints such as PD-1, BTLA, and TIM-3, resulting in increased TNF-α and IL-2 expression, thereby presenting new therapeutic avenues (95). A major challenge in immune checkpoint inhibitor (ICI) therapy is the emergence of immune-related adverse events (irAEs). miR-146a plays a regulatory role in immune cells and has been shown to alleviate irAEs when administered as a mimic. In a study involving 167 patients undergoing ICI therapy, the rs2910164 SNP in the MIR146A gene was associated with reduced miR-146a expression, shorter progression-free survival, increased irAE severity, and elevated neutrophil counts (96). Collectively, these findings highlight the pivotal role of specific miRNAs in regulating immune checkpoint pathways, offering novel insights into tumor immune evasion mechanisms and presenting promising opportunities for enhancing the efficacy and safety of cancer immunotherapies through miRNA-based interventions.

## 5 Clinical potential of miRNAs

Identifying specific tissue types, and subcategories, and ensuring timely cancer diagnosis are crucial factors in managing the disease. In addition to these considerations, miRNA signatures play a significant role in cancer prognosis (97, 98). For example, miR-214, miR-21, miR-183, miR-182, and miR-224 are frequently found to be upregulated in cervical cancer cells and tissues, whereas miR-200b, miR-150, miR-187, miR-205, and miR-636 are commonly downregulated (99).

Recent advances in miRNA-based cancer therapeutics have witnessed significant clinical evaluations of novel candidates. MRX34, a synthetic miR-34a mimic, was the first-in-class miRNA therapeutic to enter clinical trials targeting various solid tumors and hematologic malignancies (NCT01829971). Despite promising preclinical efficacy, its development was halted due to severe immune-related adverse events, including patient fatalities, underscoring the critical need for improved delivery systems and risk assessment of off-target effects (100). More recently, RGLS5579, a miRNA antagonist targeting the oncogenic miR-10b, has shown promising results in preclinical studies. Although it has not yet entered clinical trials, its combination with temozolomide significantly and safely prolonged survival in an orthotopic mouse model of glioblastoma multiforme (101). The regulatory evaluation of RGLS5579 is ongoing, with current investigations focused on determining optimal dosing strategies and therapeutic windows. These findings underscore the rapid advancement of miRNA-based therapies, while also highlighting the critical challenges in translating these approaches into clinically viable cancer treatments.

Recent research on metastatic breast cancer has explored plasma concentrations of miR-10b and miR-373. These miRNAs were pivotal in detecting lymph node metastasis, underscoring their potential as prognostic biomarkers (102). Interestingly, certain individual miRNAs demonstrate significant predictive value. Research involving breast cancer patients showed that elevated miR-210 levels correlated with an increased risk of disease recurrence and a lower likelihood of recurrence-free survival. Remarkably, the diagnostic accuracy of measuring miR-210 alone was comparable to that of a 76-gene mRNA signature test (GENE76) (103, 104). A meta-analysis conducted in 2023 comprehensively evaluated many candidate miRNAs for diagnosing ovarian cancer and highlighted nine miRNAs—miR-21, miR-125, miR-141, miR-145, miR-205, miR-328, miR-200a, miR-200b, and miR-200c—that were notably elevated in the plasma or serum of ovarian cancer patients (105). Moreover, a recently identified nine-miRNA signature model, termed ImmiRSig, has shown promising capability in predicting both overall survival and recurrence-free survival in gastric cancer. This signature comprises miR-125b-5p, miR-99a-3p, miR-145-3p, miR-328-3p, miR-133a-5p, miR-1292-5p, miR-675-3p, miR-92b-5p, and miR-942-3p. It was developed through analysis of data from 389 gastric cancer patients in The Cancer Genome Atlas (TCGA). ImmiRSig effectively categorized patients into high- and low-risk groups with significantly distinct survival outcomes and was successfully validated in an independent cohort of 193 patients (106). Additionally, a six-miRNA-based signature (miR-614, miR-1197, miR-4770, miR-3136, miR-3173, and miR-4636) was identified and validated as a powerful and independent predictor of tumor deposits in colorectal cancer patients. This six-miRNA signature demonstrated superior predictive accuracy compared to conventional clinicopathologic models and holds the potential for improving preoperative risk stratification and clinical decision-making (107). To further illustrate the translational potential of miRNA-based therapies, **Table 1** provides an overview of some selected clinical trials of miRNA therapies in solid tumors.

**Table 1:** Summary of selected ongoing clinical trials investigating miRNA therapeutics in solid tumors.

Trial ID	miRNA Target	Cancer Type	Phase	Patient Cohort	Status	Key Findings/Outcomes
**NCT04675996**	miR-193a-3p mimic (INT-1B3)	Advanced solid tumors	I/Ib	Patients with advanced malignancies	**Terminated**	First-in-human, multi-center, open-label trial sponsored by InteRNA. Aimed to assess safety, PK/PD, and preliminary efficacy of INT-1B3. Terminated due to insufficient funding.
**NCT01829971**	MRX34 (miR-RX34 liposomal injection)	Primary liver cancer, SCLC, lymphoma, melanoma, multiple myeloma, RCC, NSCLC	I	Patients with unresectable primary liver cancer or advanced/metastatic solid tumors or hematologic malignancies	**Terminated**	The trial was terminated due to five immune-related serious adverse events.
**NCT02862145**	miR-34 mimic (MRX34)	Advanced Melanoma	I/Ib	Melanoma patients with biopsy-accessible lesions	**Withdrawn**	Pharmacodynamics study of MRX34, a liposomal miR-34 mimic. Planned serial biopsies and blood sampling. The trial withdrew after 5 immune-related serious adverse events were reported in a prior Phase I study (NCT01829971).
**NCT02369198**	miR-16 mimic (TargomiRs)	Malignant Pleural Mesothelioma (MPM), Non-Small Cell Lung Cancer (NSCLC)	I	Patients with recurrent MPM and advanced NSCLC failing standard therapy	**Completed**	First-in-human trial of EGFR-targeted EDV-delivered miR-16 mimic (TargomiRs). Assessed safety and dose escalation in patients with limited treatment options. Doses ranged from 1 to 5 billion minicells weekly or biweekly. Demonstrated feasibility and potential for miRNA delivery using bacterial minicell-based system.
**NCT04811898**	LNA-i-miR-221	Refractory multiple myeloma and advanced solid tumors (including hepatocarcinoma)	I	Adult patients (≥18 years) with refractory MM or advanced solid tumors	**Completed**	Monocentric, open-label, dose-escalation study assessing safety, MTD, and RP2D of LNA-i-miR-221 using LNA technology. 5 dose cohorts, IV bolus on days 1–4 of the 28-daycycle. The study confirmed safety/tolerability and collected PK/efficacy biomonitoring data.
**NCT02580552**	miR-155 inhibitor (Cobomarsen/MRG-106)	CTCL (MF subtype), CLL, DLBCL (ABC subtype), ATLL	I	Patients with various lymphomas and leukemias	**Completed**	Phase 1 dose-ranging trial by miRagen Therapeutics evaluating safety, tolerability, PK, and efficacy of cobomarsen. Weekly subcutaneous, IV, or intratumoral injections. Demonstrated feasibility of targeting miR-155 in hematologic malignancies.
**NCT03713320**	miR-155 inhibitor (Cobomarsen/MRG-106)	CTCL (Mycosis Fungoides subtype)	II	126 patients randomized to cobomarsen or vorinostat	**Terminated**	Phase 2 randomized trial cobomarsen IV cobomarsento oral vorinostat. Assessed skin lesion severity and disease progression. Terminated early for business reasons, not due to safety or efficacy concerns. Included crossover arm for progression under vorinostat.
**NCT05908773**	miR-10b inhibitor (TTX-MC138-NODAGA-Cu64)	Advanced metastatic solid tumors	0 (Microdose)	12 patients with radiographically confirmed metastases	**Completed**	Phase 0 single-arm PET-MRI study to evaluate delivery and biodistribution of radiolabeled miRNA therapeutic TTX-MC138-NODAGA-Cu64. Demonstrated successful targeting of metastases and feasibility of image-guided miRNA delivery in humans.
**NCT06260774**	miR-10b inhibitor (TTX-MC138)	Advanced solid tumors	I/II	Adults with advanced solid tumors enrolled in multicenter dose-escalation and expansion cohorts	**Recruiting**	Open-label study delivering a single IV infusion of TTX-MC138 on Day 1 of each 28-day cycle. Dose-escalation aims to identify MTD/RP2D; the expansion phase will assess safety, PK/PD, and preliminary antitumor activity. Treatment continues until DLT, progression, or withdrawal.
**NCT02855268**	miR-21 inhibitor (Lademirsen/SAR339375)	Alport Syndrome	II	Patients with genetically confirmed Alport syndrome	**Terminated (futility)**	Placebo-controlled study; terminated following futility analysis. No unexpected safety were issues reported. Evaluated renal function preservation, PK/PD, and ADA formation.

Bold values indicate clinical trials with significant outcomes or key identifiers, such as Trial ID, that are critical for referencing specific miRNA therapeutic studies or highlighting trials with notable findings, such as successful targeting of metastases, feasibility of miRNA delivery, or termination due to adverse events or insufficient funding.

## 6 MicroRNAs used in some cancers

### 6.1 Pancreatic cancer

Pancreatic cancer (PC) exhibits the most dismal survival rates among all cancer types, with less than 1% overall 10-year survival and a mere 3% overall 5-year survival, as evidenced by data from patients in England and Wales (108). Despite significant advancements in cancer therapies, PC survival rates have remained stagnant over the past four decades (108). A fundamental contributor to this bleak scenario is the advanced stage at which PC is typically diagnosed, often accompanied by extensive metastatic lesions in the liver (109).

Certain miRNAs have been identified as oncogenic drivers in pancreatic cancer, playing crucial roles in tumor initiation and metastasis. These miRs function by suppressing genes that regulate critical cell cycle transitions, thereby promoting abnormal cell proliferation and disrupting mRNA translation. miR-212, is upregulated in pancreatic ductal adenocarcinoma (PDAC) cells, fueling cancer cell proliferation. Experimental studies involving the transfection of miR-212 mimics or inhibitors into PDAC cells have elucidated its oncogenic role. Notably, miR-212 targets PTCH1, a gene whose expression it modulates. This regulatory action, mediated by miR-212, enhances the metastatic potential of PDAC cells, driving their progression and transformation (109).

Additionally, miR-221-3p, another upregulated miR, has been implicated in PDAC. In SW1990 cells, miR-221-3p promotes cell proliferation while inhibiting apoptosis (110). Conversely, miR-128 levels are reduced in pancreatic cancer tissues compared to adjacent non-cancerous tissue (111). A study examining the role of miR-128 employed multiple techniques such as colony formation assays, flow cytometry to detect apoptotic cells, western blotting, and qRT-PCR. The findings demonstrated that miR-128 inhibits pancreatic cancer cell proliferation by targeting MDM4, a member of the double minute family that negatively regulates the tumor suppressor protein p53. By this pathway, miR-128 promotes apoptosis in cancer cells, highlighting its potential as a therapeutic target (112).

### 6.2 Colorectal cancer

Colorectal cancer (CRC) stands as the second most common cause of cancer-related deaths globally, marked by its high mortality rate and increasing incidence (113). Despite advancements in early screening, diagnosis, and prognostic tools for colorectal cancer, miRNA is emerging as a promising biomarker for evaluating its progression. Reduced expression of miRNA clusters is often linked to the onset and advancement of colorectal cancer.

In CRC cells, increased expression of the sirtuin 1 (SIRT1) protein results in decreased promoter activity, leading to the suppression of transcription of the miR-15b/16-2 cluster. Moreover, mature miR-15/16 molecules are sequestered by elevated levels of competing endogenous RNAs (ceRNAs), specifically sponge-long non-coding RNAs, in certain CRC cells (114, 115). Both *in vitro* and *in vivo* studies have confirmed the tumor-suppressive roles of the miR-15/16 clusters in CRC (116, 117). These miRNAs commonly target molecules such as cyclin B1 and transcription factor AP-4, which are critical regulators of EMT (118). Experimental overexpression of miR-15 suppresses CRC cell proliferation by targeting the anti-apoptotic protein BCL2 (119). Similarly, miR-16 overexpression decreases CRC cell growth and viability by downregulating the KRAS proto-oncogene, a key oncogenic GTPase, demonstrated in both *in vitro* and *in vivo* models (119). Additionally, an inverse relationship between miR-16 expression and the levels of vascular endothelial growth factor (VEGF) receptor and the MYB proto-oncogene has prognostic significance in CRC patients (120).

The expression levels of miR-99a and miR-99b correlate with the amount of mTOR protein in CRC cell lines (121). Overexpression of miR-125 family members promotes apoptosis in CRC cells by targeting anti-apoptotic factors such as BCL2, other BCL2 family proteins like BCL2L12, and the Mcl-1 gene (122). Furthermore, increased miR-125a levels inhibit angiogenesis and metastasis in CRC by targeting genes including VEGFA, SMURF1, and CREB5 (123, 124). Members of the let-7 family contribute to cell cycle arrest and reduced proliferation by targeting genes such as PHRF2, the RTKN, IGF1, and MYC (125–127). Specifically, upregulation of let-7c or let-7e suppresses metastatic potential by downregulating MMP11, PBX3, and DCLK1 proteins (128, 129). Additionally, modulation of these targets by let-7 family members enhances the sensitivity of CRC cells to chemotherapy and radiotherapy (130).

## 7 MicroRNA-based therapeutics

MiRNA-based therapeutics comprise two main categories: miRNA mimics and miRNA inhibitors (antimiRs) (92). MiRNA mimics are synthetic small RNA molecules engineered to bind to specific miRNA sequences, restoring reduced miRNA expression in particular diseases (131). Conversely, miRNA inhibitors are used to suppress the expression of oncogenic microRNAs (132). This therapeutic strategy shows promise in cancer treatment due to established connections between abnormal gene expressions and tumorigenesis, making it a viable option for combating cancer (131, 133). Circulating miRNAs can be readily obtained with minimal harm. Moreover, a wide range of potentially valuable miRNA biomarkers has been identified as stable in healthy individuals. While cell-free miRNAs isolated from serum and plasma are commonly used as circulating miRNA biomarkers, other bodily fluids like saliva and urine also serve as relevant sources of these miRNAs (134). Overexpression of miR-186-5p has been observed in tumor tissue, urine, and blood samples from individuals with bladder cancer (135). Several miRNAs, including miR-210-3p, were found to be elevated in the urine of patients with transient cell carcinoma, indicating their potential to improve cancer detection (136). Polymerase chain reaction (PCR) remains the primary method for assessing circulating miRNA levels. PCR is crucial for amplification, significantly enhancing the distinctions between samples, even those with subtle differences. Consequently, this detection technique renders circulating miRNAs the most sensitive biomarkers available. MiRNAs are generated promptly and adaptively in response to internal or external stimuli, enabling their real-time and dynamic monitoring throughout various stages of progression, from tumor initiation to metastasis and beyond (137, 138).

Therapeutic modulation of miRNA expression offers considerable potential for disease prevention and treatment. Several approaches have been developed to target miRNAs, including small molecules that influence miRNA transcription and processing, as well as inhibitors that block miRNA activity (139). Among these, inhibition of miRNA function stands out as a critical strategy in novel therapeutic developments. Antisense oligonucleotides have emerged as a sophisticated method to achieve this by directly binding to miRNAs within the RISC, thereby preventing their interaction with target mRNAs (140, 141). As a result, antisense-based inhibition of miRNAs is poised to play a pivotal role in the advancement of future therapeutic interventions (142).

Among the different strategies for targeting miRNAs, antisense oligonucleotides stand out as the most advanced approach. Specific types of these oligonucleotides bind directly to miRNAs within the RISC, effectively preventing their interaction with target mRNAs, as demonstrated in various studies (142). Notably, the miRNA-targeting oligonucleotide SPC3649 (Miravirsen) has shown encouraging therapeutic potential in preclinical and clinical investigations (143).

Another promising strategy involves using miRNA sponges, which contain multiple binding sites to simultaneously inhibit several miRNAs. However, most research on these sponges has so far been limited to animal models (143, 144). Additionally, circular RNAs (circRNAs) have been recognized as natural miRNA sponges in certain tissues, contributing to the regulation of miRNA activity (145). A different technique, known as miRNA masking, employs oligonucleotides designed to bind the miRNA recognition sites within the 3′-UTR of target mRNAs, thereby blocking miRNA binding and preventing gene silencing (146). Furthermore, some pharmacological agents have been found to influence miRNA expression and the complex signaling pathways involved in miRNA biogenesis (147). For example, azobenzene has been shown to reduce miR-21 levels by inhibiting its precursor processing inside cells (148). While these approaches hold considerable promise, additional studies are required to optimize their delivery systems and improve their therapeutic effectiveness.

In addition to inhibiting miRNAs, another therapeutic approach involves the use of miRNA mimics to restore the function of miRNAs that are downregulated in disease conditions. Synthetic miRNA mimics can be introduced to increase the levels of specific miRNAs, thereby compensating for their reduced expression (149). For instance, miR-34, a well-known tumor suppressor miRNA, is often found at decreased levels in several cancers, including breast and colon cancer. Treatment with miR-34 mimics has shown the potential to inhibit tumor growth and proliferation, highlighting its promise as a therapeutic strategy (150).

Twenty-eight patients received a single subcutaneous injection of RG-101. After 4 weeks, all patients exhibited a significant reduction in viral load, and RG-101 was generally well tolerated. Remarkably, three patients maintained undetectable HCV RNA levels even 76 weeks following a single dose. However, some patients experienced viral rebound after 12 weeks, which correlated with the emergence of resistance mutations within the miR-122-binding sites in the 5′-untranslated region of the HCV genome (151). Despite these promising antiviral effects, the trial was halted due to elevated bilirubin levels detected in patients’ blood. RG-101 induced unintended side effects, including impaired transport of conjugated bilirubin and disruption of baseline bilirubin transport, likely due to its preferential uptake by hepatocytes, leading to hyperbilirubinemia. This line of research, targeting miR-122 in HCV-infected patients, is similar to the Miravirsen approach. Notably, locked nucleic acid (LNA)-antisense-based therapies like Miravirsen have demonstrated an advantage with fewer side effects compared to other anti-miRNA strategies. Furthermore, no escape mutations have been reported in patients treated with Miravirsen, whereas such mutations arose during RG-101 therapy, despite both treatments targeting endogenous miR-122. Given that both RG-101 and RG-125 trials—using N-acetylgalactosamine (GalNAc)-conjugated anti-miRNAs—were suspended, it is tempting to speculate that the GalNAc conjugation method may contribute to the observed adverse effects. To facilitate a comprehensive understanding of the diverse therapeutic modalities, **Table 2** summarizes the mechanisms, benefits, and limitations of currently investigated miRNA-based approaches.

**Table 2:** miRNA-based therapeutic strategies: mechanisms, applications, and challenges.

Therapeutic approach	Mechanism of action	Potential applications	Examples/Case studies	Challenges and limitations
miRNA Mimics	Replacement or enhancement of downregulated tumor-suppressor miRNAs	Inhibition of tumor growth in cancers with suppressed miRNA expression	miR-34 mimic therapy in breast and colon cancer (150)	Delivery efficiency, intracellular stability, off-target effects
AntimiRs (Inhibitors)	Inhibition of oncogenic miRNAs using antisense oligonucleotides	Suppressing miRNAs that promote tumorigenesis or viral replication	Miravirsen (SPC3649) targeting miR-122 in HCV (143)	Resistance mutations, side effects (e.g., hyperbilirubinemia in RG-101), and delivery refinement needed
miRNA Sponges	Sequestration of multiple miRNAs via transcripts with multiple binding sites	Simultaneous inhibition of multiple miRNAs, mainly tested in animal models	circRNAs acting as natural sponges (145)	Limited human studies, inefficient delivery
miRNA Masking	Blocking miRNA binding sites within the 3′-UTR of target mRNAs using masking oligos	Restoring target gene expression repressed by specific miRNAs (e.g., CFTR in cystic fibrosis)	PNA-based masking of miR-145-5p binding sites in CFTR 3′-UTR, leading to increased CFTR mRNA/protein levels in Calu-3 and CFBE41o cells; enhanced efficacy with VX-770/VX-809 (152)	Requires precise sequence design, potential for off-target interactions
Drug-Based miRNA Modulation	Inhibiting miRNA biogenesis or processing pathways via small molecules	Targeted downregulation of pathogenic miRNAs	Azobenzene downregulating miR-21 (148)	Nonspecific effects, potential cytotoxicity

## 8 Conclusion

In summary, the review provides a comprehensive understanding of the potential treatment of solid tumors through microRNA targeting. It emphasizes substantial advancements in understanding miRNA biology and its implications for cancer therapy. Despite challenges, the promising results from both preclinical and clinical studies highlight the transformative potential of miRNA-based treatments. As ongoing research reveals more about miRNA networks, these innovative therapies are expected to play a crucial role in shaping the future of cancer treatment.
